# Public Health Nurses’ Competence Related to Long‐Term Breastfeeding in the Context of Maternity and Child Health Clinics

**DOI:** 10.1111/phn.13457

**Published:** 2024-10-16

**Authors:** Oona Ojantausta, Niina Pöyhönen, Marja Kaunonen, Heini Huhtala, Riikka Ikonen

**Affiliations:** ^1^ Faculty of Social Sciences Health Sciences Tampere University Tampere Finland; ^2^ Department of Nursing Science Faculty of Health Sciences University of Eastern Finland Kuopio Finland; ^3^ Pirkanmaa Wellbeing Services General Administration Tampere Finland

**Keywords:** lactation, maternal‐child health, public health nursing competencies

## Abstract

**Aim:**

To explore public health nurses' competence (namely knowledge, skills, and attitudes) in relation to long‐term breastfeeding and their experience of the need for additional training on the subject.

**Design:**

The study design was quantitative, descriptive, and cross‐sectional.

**Sample:**

Public health nurses (*n* = 270).

**Methods:**

Data were collected with the Long‐Term Breastfeeding Competence Scale (LBCS) online survey. Data analysis was done with Spearman's correlation analysis and binary logistic regression analysis.

**Results:**

Slightly more than half of the respondents had a good level of knowledge and skills. The majority had a baseline positive attitude toward long‐term breastfeeding, but the attitude became more negative as the age of the breastfed child increased. Better competence was associated with younger age, parenthood, an additional degree in midwifery, and breastfeeding specialist certification. Knowledge and skills, and attitudes revealed a high correlation: the higher the knowledge and skills level, the more positive attitudes. Respondents with better knowledge and skills experienced more often the need for additional training on the subject.

**Conclusions:**

This study addresses that public health nurses lack competence in relation to long‐term breastfeeding. This may compromise the quality of breastfeeding guidance for families in healthcare settings.

## 1 Background

Mothers breastfeeding their toddlers describe that breastfeeding is important for the child even after infancy and that one of their main motivations for long‐term breastfeeding is that their child has clearly expressed a wish to continue it (Dowling and Pontin 2017; Thompson, Topping, and Jones 2020). Breastmilk can provide one‐third of a toddler's daily energy intake alongside a significant amount of vitamins and minerals (Lackey et al. 2021). Children breastfed for a longer period have lower infectious morbidity and mortality rates, a lower prevalence of obesity, and a higher IQ level later in life. Long‐term breastfeeding reduces the risk of childhood leukemia and mothers’ breast cancer risk. Although breastfeeding should be continued for at least 2 years to achieve all its benefits (Victora et al. 2016), health professionals’ support for long‐term breastfeeding is often lacking (Jackson and Hallam 2020).

Breastfeeding rates differ across the world; the proportion of children breastfed until the age of 2 years is 45% globally, while the global goal is 60% (United Nations Children's Fund [UNICEF] and World Health Organization [WHO] 2022). WHO recommends that all children should be exclusively breastfed for 6 months and that breastfeeding should continue for up to 2 years or beyond alongside complementary foods (WHO 2023). The factors affecting breastfeeding duration include demographic, socioeconomic, and national ones, such as paid parental leaves, workplace policies, and public support (Lubold 2019). This study was performed in the context of Finnish healthcare, where maternity and child health clinics form the basis for breastfeeding promotion, along with maternity hospitals. Contrary to the WHO recommendations, in Finland it is recommended to breastfeed exclusively until the age of 4–6 months and to continue breastfeeding along with solid foods until the age of 1 year or longer if the family so desires (Finnish Institute for Health and Welfare 2019). No statistical data on breastfeeding beyond 12 months is available in Finland. However, more than half (58%) of children aged 12 months are still breastfed (Ikonen et al. 2020), which is the highest rate in comparison to other Nordic and Baltic countries (Hörnell and Lagström 2024). Although Finland can be considered a breastfeeding‐friendly country with full‐paid parental leave, a supportive health policy, and an accepting breastfeeding culture, a conflict emerges between long‐term breastfeeding and the cultural norm, and Finnish mothers feel that both healthcare (Säilävaara 2023) and society have become alienated from breastfeeding beyond 12 months (Ojantausta and Kaunonen 2021).

Although breastfeeding duration should be left for each mother and child to decide, it is influenced by several factors, including encounters with health professionals (Cisco 2017; Jackson and Hallam 2020). In Finland, mothers perceive health professionals’ attitudes toward long‐term breastfeeding as unpredictable and professional‐bound. Mothers feel that health professionals have incorrect information regarding long‐term breastfeeding and that professionals’ personal attitudes and views influence the way they encounter breastfeeding mothers. (Ojantausta and Kaunonen 2021; Säilävaara 2023) When mothers receive incorrect information about long‐term breastfeeding from health professionals, their trust in healthcare may suffer. This leads the mothers to seek breastfeeding‐related guidance from peer support groups instead (Jackson and Hallam 2020; Ojantausta and Kaunonen 2021; Säilävaara 2023). Coherent support for Finnish families has been found lacking as breastfeeding beyond 12 months is not included in the Finnish guidelines of maternity and child health clinics for breastfeeding counseling (Hakulinen, Otronen, and Kuronen 2017).

This study explores the long‐term breastfeeding‐related competence of Finnish public health nurses working in maternity and/or child health clinics. The United States Breastfeeding Committee defines the core competencies for breastfeeding support as “*the minimal knowledge, skills, and attitudes necessary for health professionals from all disciplines to provide patient care that protects, promotes, and supports breastfeeding*” (United States Breastfeeding Committee [USBC] 2010). In addition to concrete breastfeeding counseling content, competence requires recognizing the limits of one's knowledge and skills and understanding the effect one's personal values and biases may have on encounters with families (USBC 2010). In this study, health professionals’ breastfeeding competence refers to their knowledge, skills, and attitudes in relation to providing support and counseling for long‐term breastfeeding.

Health professionals’ long‐term breastfeeding‐related knowledge and skills vary and are influenced by their attitudes (Ojantausta, Pöyhönen, and Ikonen 2023), which become more negative as the breastfed child's age increases (Cockerham‐Colas, Geer, and Benker 2012; Cisco 2017; Goldbort, Hitt, and Zhuang 2023). Health professionals’ own short or lacking breastfeeding experience has a negative effect on their long‐term breastfeeding‐related attitudes (Baranowska et al. 2019; Prokop, Meedya, and Sim 2021). Furthermore, health professionals with children of their own have a more positive attitude toward long‐term breastfeeding than those without children (Cockerham‐Colas, Geer, and Benker 2012; Wright and Hurst 2018). These varying attitudes compromise the implementation of breastfeeding recommendations and health promotion. The need for support and evidence‐based information continues when breastfeeding is continued beyond infancy (Dowling and Brown 2013; Blixt, Johansson, and Hildingsson 2019) and to provide appropriate counseling, professionals must understand how the needs for support related to long‐term breastfeeding differ from those concerned with breastfeeding a baby (Tchaconas, Keim, and Heffern 2018; Thompson, Topping, and Jones 2020).

This study defines long‐term breastfeeding, also referred to as extended breastfeeding, breastfeeding beyond infancy, and prolonged breastfeeding, as breastfeeding beyond 12 months of age. Long‐term breastfeeding is a relatively unstudied subject and can be perceived as unusual by health professionals and people in general (Stearns 2011; Brockway & Venturato 2016). Previous studies have shown that health professionals lack the necessary knowledge, skills, and attitudes to provide breastfeeding guidance and support for families breastfeeding beyond infancy (Cockerham‐Colas, Geer, and Benker 2012; Gavine, MacGillivray, and Renfrew 2016; Baranowska et al. 2019). There is no clear understanding of whether health professionals recognize their own competence level in relation to long‐term breastfeeding. This is the first study to explore Finnish health professionals’ competence (including knowledge, skills, and attitudes) related to long‐term breastfeeding and their experience of the need for additional training on the subject.

### 1.1 Aim

To explore public health nurses' competence (namely knowledge, skills, and attitudes) in relation to long‐term breastfeeding, and their experience of the need for additional training. The research questions are as follows:

1. To what extent do public health nurses have accurate knowledge and skills about long‐term breastfeeding?
2. What attitudes do public health nurses hold toward long‐term breastfeeding for children in the following three age groups: 1‐, 2‐, and 3‐year‐olds or older?
3. Which public health nurses’ socio‐demographic characteristics are associated with competence (namely knowledge, skills, and attitudes) and their experience of the need for additional training in relation to long‐term breastfeeding?
4. To what extent is there an association between public health nurses’ knowledge and skills, attitudes, and their experience of the need for additional training in relation to long‐term breastfeeding?

In summary, this study explores four research questions to understand public health nurses’ (1) level of knowledge and skills in relation to long‐term breastfeeding, (2) attitudes in relation to long‐term breastfeeding, (3) socio‐demographic characteristics in association with their competence, and (4) knowledge and skills in association with attitudes and their experience of the need for additional training.

## 2 Methods

### 2.1 Design

The study design was quantitative, descriptive, and cross‐sectional.

### 2.2 Setting and Sample

The participants of this study were Finnish public health nurses currently working in maternity and child health clinics. Maternity and child health clinics provide support to families during pregnancy and throughout the first 7 years of their children's lives through regular appointments. These services are provided free of charge, and nearly all Finnish children (99.5%) are using them (Hakulinen, Hietanen‐Peltola, and Jahnukainen 2022). National guidelines provide a framework for maternity and child health clinics in monitoring a child's growth and development, promoting the well‐being of families, and supporting parenting. Breastfeeding counseling is a crucial part of health promotion in this setting (Hakulinen, Otronen, and Kuronen 2017). To work in a Finnish maternity and child health clinic, a public health nurse qualification is mandatory. A significant majority (97%) of the Finnish public health nurses working in these settings have completed the WHO's 20‐hour breastfeeding counselor course (Ikonen et al. 2020). In addition, some public health nurses may have additional qualifications, such as breastfeeding specialist or midwifery training.

The request to participate in the study was sent to all members of Finnish public health nurses’ labor unions (*n* = 10,953) via email between April and June 2023. The request was also distributed through the social media channels of the labor unions. Only some of the public health nurses who belong to these labor unions are employed in maternity and child health clinics. However, no information is available about the exact number of public health nurses working in such settings in Finland. Only those participants who responded to both dimensions (Knowledge and Skills, and Attitudes) of the instrument were included in the study and due to this, 32 participants were excluded from the analyses, making the final sample size *n* = 270. In the case of single missing values, imputation was not done.

### 2.3 Data Collection Tool

The Finnish‐language version of the Long‐term Breastfeeding Competence Scale (LBSC) was used for assessing the public health nurses’ competence (namely their knowledge, skills, and attitudes) in relation to long‐term breastfeeding. The LBCS was formed based on the Extended Breastfeeding Knowledge and Attitudes scale (Cockerham‐Colas, Geer, and Benker 2012) and the results of a systematic review (Ojantausta, Pöyhönen, and Ikonen 2023). The LBCS was piloted and validated using a panel of experts, Rasch analysis, and exploratory factor analysis. The LBCS met the criteria set for a new instrument (Pöyhönen et al. 2024).

The LBCS consisted of two dimensions: Knowledge and Skills, and Attitudes. The Knowledge and Skills dimension included nine items with True/False/Don't Know response options. The items measured the respondents’ knowledge and skills in the contexts of breastfeeding statistics, breastfeeding during pregnancy, night‐time breastfeeding, national breastfeeding recommendations, economic benefits, nutritional value, health benefits for the mother and the child, and relationship benefits.

The Attitude dimension consisted of 22 items measuring the respondents’ attitudes toward long‐term breastfeeding in breastfed children's age categories 1‐, 2‐, and 3‐year‐olds or older. The first attitude item explored the respondent's opinion about the normality of breastfeeding a child aged over 1 year and the remaining 21 items measured the respondent's attitudes by age group so that there were seven statements concerning each of the three age groups. In these seven items, attitudes were explored from the perspectives of acceptability, embarrassment, health effects on the mother's and child's physical and psychological health, and encouragement to stop breastfeeding. The response options for the attitude items were on a 5‐point Likert scale (*completely agree, somewhat agree, neither agree nor disagree, somewhat disagree, completely disagree*). The English translation of the LBCS is provided in the Supporting Material.

In addition, the questionnaire collected socio‐demographic background information about the respondents, including their age, gender, professional education, training related to breastfeeding counseling as a breastfeeding counselor (WHO 20‐hour training course) or a breastfeeding specialist (International Board Certified Lactation Consultant [IBCLC] or breastfeeding counselor trainer), work unit, work experience in years, and personal parental and breastfeeding background. In addition, the respondents were asked about their participation in breastfeeding training during the previous 5 years, participation in training related to breastfeeding beyond 12 months, and their experience of the need for additional training on the subject.

### 2.4 Data Analysis

A descriptive analysis of the data was performed. The variables were described through frequencies and percentages. The normality of the distributions of the variables was examined using the Kolmogorov–Smirnov test. Due to the skewness of the variables, medians and quartiles (*Q*_1_–*Q*_3_) were reported. The socio‐demographic background variables gender, working unit, and the respondent's own breastfeeding experience were excluded because, due to unanimous responses, these variables did not differentiate the respondents. Background information on the respondents’ participation in WHO's 20‐Hour Training Course was excluded from the analysis, as it conflicted with national statistical data (Ikonen et al. 2020). Cross‐tabulation and Chi‐square tests were used to detect associations between background variables. Background variables that had a statistically significant association (*p* < 0.05) with other background variables were excluded from the multivariable regression analyses. This led to excluding the respondents’ years of working experience, participation in any breastfeeding training, and participation in breastfeeding training regarding breastfeeding beyond 12 months. Fisher's test was used to statistically evaluate differences in the respondents’ answers in the attitude items concerning the breastfed children's age categories (1‐, 2‐, and 3‐year‐olds or older). The IBM SPSS Statistics v. 28.0 software was used for the statistical analyses in this study.

Sum variables were formed to describe the levels of competence. Binary logistic regression analysis was used with the ENTER method to estimate odds ratios (OR) and 95% confidence intervals (% CI) for the level of knowledge and skills, level of attitude, and the background variable describing public health nurses’ experience of the need for additional training in association with socio‐demographic characteristics. The goodness of fit of the model was assessed using the Hosmer–Lemeshow test. The associations between knowledge and skills, attitudes, and experience of the need for additional training were analyzed using Spearman's rank correlation and binary logistic regression analysis. The correlation coefficient was interpreted as low (*r* ≤ 0.30), moderate (*r* = 0.30−0.50), or high correlation (*r* = > 0.50). To handle missing data, different deletion techniques were employed based on the type of analysis conducted. The pairwise deletion method was utilized for the descriptive analyses and Fisher's exact test, resulting in a sample size of *n* = 270. For the binary logistic regression analysis, we applied the listwise deletion method, which ensured a consistent sample across these analyses. This resulted in a sample size of *n* = 256. A statistical significance level of < 0.05 was used in all the analyses.

#### 2.4.1 Knowledge and Skills Dimension

Nine items in the LBCS measured the respondents’ knowledge and skills regarding breastfeeding beyond 12 months. Two sum variables were formed based on these knowledge and skills items. The items were classified as dichotomous variables (1 = *correct answer*, 0 = *incorrect answer* or *don't know*). The first sum variable was cumulative, as it was formed to describe variation in knowledge and skills points (range 0−9 points).

Rasch analysis (Boone 2016), used when validating the LBCS, was utilized in assessing the difficulty of the items and the knowledge level of the respondents. Wright's map results suggested that three of the knowledge and skills items stood out as being perceived as more difficult than the other six items. This information was used in defining the cut‐off point for a good and an insufficient level of knowledge and skills. The cut‐off point for a good level of knowledge and skills was considered to be seven points (0–6 points being an insufficient level of knowledge, 7–9 points being a good level of knowledge). Based on this, the second sum variable was formed to distinguish between a good and insufficient level of knowledge and skills. The sum variable was used to explore the associations between the level of knowledge and skills and other variables.

#### 2.4.2 Attitude Dimension

Attitude items were reclassified by combining *completely agree* and *somewhat agree* into *agree*, as well as *completely disagree* and *somewhat disagree* into *disagree* to describe the distribution of responses. For the rest of the analysis, the 22 attitude items were classified as dichotomous variables (1 = *positive response*, 0 = *negative or neutral response*). The response option “Neither agree nor disagree” was interpreted as a neutral response. Four sum variables were formed based on the attitude items. The first three attitude sum variables were formed to describe the attitude toward breastfeeding in three age groups of breastfed children: 1‐, 2‐, and 3‐year‐olds or older. The attitude sum variables for the age groups were formed respectively a positive attitude was deemed as equal to the respondent answering positively to the item on whether breastfeeding beyond 1 year of age was normal, as well as to all seven items related to this particular age group (0–7 points meaning a negative or neutral attitude, 8 points signifying a positive attitude).

The final attitude sum variable was formed to describe the respondents’ baseline attitude toward long‐term breastfeeding and to explore the associations between the baseline attitude and other variables. The cut‐off point for the baseline attitude toward long‐term breastfeeding was considered to be 16 points (0–15 points signifying a negative or neutral attitude, 16–22 points signifying a positive attitude). This was considered equal to a positive response to the item on whether breastfeeding beyond 1 year of age was normal, to all 14 items considering the breastfeeding of a 1‐ and a 2‐year‐old child and to one item considering the breastfeeding of a 3‐year‐old or older child. This was considered to be in line with international breastfeeding recommendations and to show a positive attitude toward the continuation of breastfeeding even beyond 2 years. In addition, public health nurses are expected to have an active approach to promoting breastfeeding and to individually support families and their decisions regarding breastfeeding.

### 2.5 Ethical Considerations

An affirmative statement for the research project was obtained from the Ethics Committee of the Tampere Region, Finland on 9 ^h^ May 2022 (statement no. 51/2022).

## 3 Results

### 3.1 Characteristics of the Sample

The respondents (*n* = 270) were public health nurses, of whom 78.4% worked in combined maternity and child health clinics where, in addition to children, they provided services to pregnant mothers. In addition, 19% of the nurses worked in child health clinics, and 2.6% in maternity clinics. The median age of the respondents was 37 years (*Q*_1_–*Q*_3_ = 32−45) and the median work experience was 7.0 (3−14) years. Most of the respondents (85.2%) had a child/children of their own, and 99.1% of the nurses who were parents had breastfed their child. All socio‐demographic characteristics are described in Table 1.

**TABLE 1:** Socio‐demographic characteristics of the public health nurses (*N* = 270).

Characteristic	*n*	%
Age group		
29 years and younger	42	15.6
30−39 years	109	40.4
40−49 years	67	24.8
50 years and older	41	15.2
Missing data	11	4.1
Gender		
Female	266	98.5
Rather not say	2	0.7
Missing data	2	0.7
Profession		
Public health nurse	269	99.6
In addition, midwife	38	14.1
Education related to BF counseling		
Breastfeeding counselor	175	64.8
Breastfeeding specialist	20	7.4
Working unit		
Maternity and child health clinic	211	78.1
Child health clinic	51	18.9
Maternity clinic	7	2.6
Missing data	1	0.4
Years of work experience		
< 2 years	29	10.7
2−5 years	73	27.0
6−10 years	60	22.2
11−20 years	72	26.7
> 20 years	25	9.3
Missing data	11	4.1
Own experiences		
Own child/children	230	85.2
Own BF experience	228	99.1
No child/children	40	14.8
BF education		
Participation in any BF education during the previous 5 years	220	81.5
Participation in education related to BF beyond 12 months	48	17.8
Experience of the need for additional training related to BF beyond 12 months	195	72.2

*Note*: Breastfeeding counselor = WHO 20 h training course, breastfeeding specialist = IBCLC or breastfeeding counselor trainer.

Abbreviations: BF, breastfeeding; IBCLC, International Board Certified Lactation Consultant.

### 3.2 Public Health Nurses’ Knowledge and Skills

The median point of the nine knowledge and skills items was 7 (6−8). Slightly more than half of the public health nurses (*n* = 151, 55.9%) had a good level of knowledge and skills. The distribution of the respondents’ correct answers for each knowledge and skills item is presented in Table 2.

**TABLE 2:** Public health nurses’ (*N* = 270) competence in knowledge and skills items.

	Correct answer	
Knowledge and skills item (correct answer)	*n*	%
In Finland, there are _____ children approaching the age of one who are breastfed (58%)	43	15.9
The mother should stop breastfeeding the older child after becoming pregnant (No)	251	93.0
The mother should stop nighttime breastfeeding when the child reaches 1 year of age (No)	157	58.1
In Finland, it is recommended to stop breastfeeding when the child reaches 1 year of age (No)	243	90.0
Breastfeeding beyond 12 months is economically beneficial for the family (Yes)	118	43.7
Breast milk is good nutrition for a child over 1 year old (Yes)	220	81.5
Women who have breastfed for more than a year have a ____ risk of premenopausal breast cancer (lower)	243	90.0
Children breastfed for more than a year have a ____ risk of childhood and adult obesity (lower)	238	88.1
The effect of breastfeeding beyond 12 months on the attachment relationship between parent and child is (positive)	235	87.0

### 3.3 Public Health Nurses’ Attitudes

**FIGURE 1:**
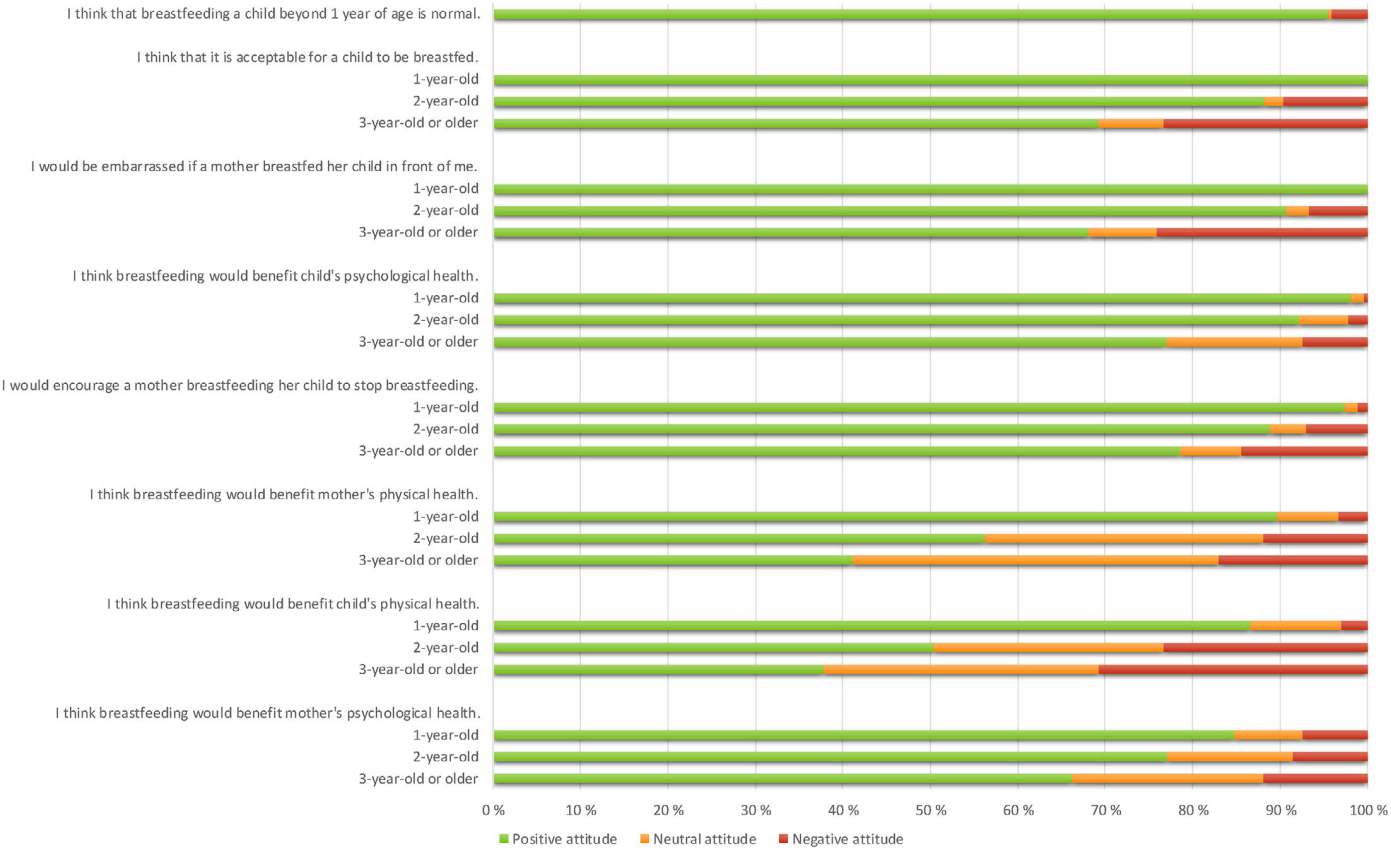
Distribution of attitudes toward long‐term breastfeeding divided by age groups: 1‐, 2‐, and 3‐year‐olds or older. [Colour figure can be viewed at wileyonlinelibrary.com]

The respondents’ attitudes toward long‐term breastfeeding became less positive in each of the 22 attitude items as the breastfed child's age increased (Figure 1). Table 3 presents the respondents’ responses to the attitude items across three age groups of breastfed children: 1‐, 2‐, and 3‐year‐olds or older. The table also displays the results of Fisher's exact test regarding the differences in the responses between these age groups of breastfed children.

**TABLE 3:** Public health nurses’ (*N* = 270) competence in attitude items in age groups 1‐, 2‐, and 3‐year‐olds or older.

	Age of the breastfed child						
	1 year		2 years		3 years or older		
Attitude item	*n*	%	*n*	%	*n*	%	*p* value^a^
I think that breastfeeding a child beyond 1 year of age is normal.							
Agree	258	95.6					
Disagree	11	4.1					
Neither agree or disagree	1	0.4					
I think that it is acceptable for a child to be breastfed							< 0.001^b^
Agree	270	100.0	238	88.1	187	69.3	
Disagree	0	0	26	9.6	63	23.3	
Neither agree or disagree	0	0	6	2.2	20	7.4	
I would be embarrassed if a mother breastfed her child in front of me							< 0.001^b^
Agree	0	0	18	6.7	65	24.1	
Disagree	270	100	245	90.7	184	68.1	
Neither agree or disagree	0	0	7	2.6	21	7.8	
I would encourage a mother breastfeeding her child to stop breastfeeding							< 0.001
Agree	3	1.1	19	7.0	39	14.4	
Disagree	263	97.4	240	88.9	212	78.5	
Neither agree or disagree	4	1.5	11	4.1	19	7.0	
I think breastfeeding would benefit a child's physical health							< 0.001
Agree	234	86.7	136	50.4	102	37.8	
Disagree	8	3.0	63	23.3	83	30.7	
Neither agree or disagree	28	10.4	71	26.3	85	31.5	
I think breastfeeding could cause psychological harm to the child							< 0.001
Agree	1	0.4	6	2.2	20	7.4	
Disagree	265	98.1	249	92.2	208	77.0	
Neither agree or disagree	4	1.5	15	5.6	42	15.6	
I think breastfeeding would benefit a mother's physical health							< 0.001
Agree	242	89.6	152	56.3	111	41.1	
Disagree	9	3.3	32	11.9	46	17.0	
Neither agree or disagree	19	7.0	86	31.9	113	41.9	
I think breastfeeding could cause psychological harm to the mother							< 0.001
Agree	20	7.4	23	8.5	32	11.9	
Disagree	229	84.8	208	77.0	179	66.3	
Neither agree or disagree	21	7.8	39	14.4	59	21.9	

^a^ Fisher's exact test.

^b^ Age groups 2 and 3 years or older included in the analysis.

The respondents’ positive attitudes toward breastfeeding varied by age group: 69.3% of the respondents had a positive attitude toward breastfeeding a 1‐year‐old, 33.3% had a positive attitude toward breastfeeding a 2‐year‐old, and 23% had a positive attitude toward breastfeeding a 3‐year‐old or older child.

The majority of the public health nurses (*n* = 191, 70.7%) had a positive attitude toward long‐term breastfeeding at baseline. The median points of the attitude items were 18 (*Q*_1_–*Q*_3_ = 151−21).

### 3.4 Socio‐Demographic Characteristics' Associations With Competence

The univariate logistic regression analysis showed an association between the respondents’ age, qualification as a breastfeeding specialist, having child(ren), and knowledge and skills. The multivariable logistic regression analysis showed that the odds of having a good level of knowledge and skills were significantly lower for the older public health nurses, and higher for the public health nurses with the breastfeeding specialist certification and for those who had a child of their own (Table 4).

**TABLE 4:** The association between the public health nurses’ (*n* = 256) socio‐demographic characteristics and their level of knowledge and skills.

Baseline characteristic	Univariate			Multivariable		
	OR	95% CI	*p* value	OR	95% CI	*p* value
Age	0.97	0.95−1.00	0.019	0.95	0.92−0.98	< 0.001
Work experience	0.97	0.94−1.00	0.077	Not entered*		
Midwife	1.35	0.65−2.81	0.426	1.39	0.64−3.04	0.409
Breastfeeding specialist	6.76	1.52−30.05	0.012	6.83	1.50−31.18	0.013
Own child	2.14	1.05−4.36	0.035	3.13	1.41−6.92	0.005
Participation in any BF education during the previous 5 years	1.20	0.64−2.36	0.574	Not entered**		
Participation in education related to BF beyond 12 months	1.88	0.95−3.74	0.071	Not entered***		

*Note*: Goodness of fit (Hosmer–Lemeshow test) *p* = 0.311.

High correlation with age *r* = 0.794, *p < 0.001.

Statistically significantly associated with IBCLC certification **p = 0.032, ***p < 0.001.

Breastfeeding specialist = IBCLC or breastfeeding counselor trainer.

Abbreviations: BF, breastfeeding; IBCLC, International Board Certified Lactation Consultant.

The univariate logistic regression analysis showed an association between an additional degree in midwifery and the level of attitude. The multivariable logistic regression analysis showed that the odds of having a positive attitude were significantly lower for older public health nurses and higher for those with an additional degree in midwifery (Table 5).

**TABLE 5:** The association between the public health nurses’ (*n* = 256) socio‐demographic characteristics and the level of attitude.

Baseline characteristic	Univariate			Multivariable		
	OR	95% CI	*p* value	OR	95% CI	*p* value
Age	0.97	0.95−1.00	0.060	0.96	0.93−0.99	0.008
Work experience	0.98	0.95−1.02	0.301	Not entered*		
Midwife	3.52	1.20−10.35	0.022	3.82	1.26−11.54	0.018
Breastfeeding specialist	7.37	0.96−56.46	0.054	6.89	0.89−53.57	0.065
Own child	1.44	0.69−3.00	0.337	1.82	0.81−4.10	0.146
Participation in any BF education during the previous 5 years	0.78	0.38−1.59	0.488	Not entered**		
Participation in education related to BF beyond 12 months	1.49	0.70−3.19	0.305	Not entered***		

*Note*: Goodness of fit (Hosmer–Lemeshow test) *p* = 0.674.

High correlation with age *r* = 0.794, *p < 0.001.

Statistically significantly associated with IBCLC certification **p = 0.032, ***p < 0.001.

Breastfeeding specialist = IBCLC or breastfeeding counselor trainer.

Abbreviations: BF, breastfeeding; IBCLC, International Board Certified Lactation Consultant.

The univariate logistic regression analysis showed an association between age, work experience, and experience of the need for additional training. The multivariable logistic regression analysis showed that the odds of experiencing the need for additional training were significantly higher for younger public health nurses and those with breastfeeding specialist certification (Table 6).

**TABLE 6:** The association between public health nurses’ (*n* = 256) socio‐demographic characteristics and their experience of the need for additional training on the subject.

Baseline characteristic	Univariate			Multivariable		
	OR	95% CI	*p* value	OR	95% CI	*p* value
Age	0.96	0.93−0.99	0.009	0.96	0.94−0.99	0.015
Work experience	0.95	0.91–.98	0.002	Not entered*		
Midwife	0.64	0.30−1.36	0.243	0.65	0.30−1.43	0.281
Breastfeeding specialist	6.52	0.85−50.00	0.071	8.33	1.06−65.69	0.044
Own child	0.62	0.26−1.48	0.281	0.78	0.31−1.96	0.596
Participation in any BF education during the previous 5 years	0.68	0.32−1.44	0.310	Not entered**		
Participation in education related to BF beyond 12 months	1.12	0.53−2.35	0.772	Not entered***		

*Note*: Goodness of fit (Hosmer–Lemeshow test) *p* = 0.673.

High correlation with age *r* = 0.794, *p < 0.001.

Statistically significantly associated with IBCLC certification **p = 0.032, ***p < 0.001.

Breastfeeding specialist = IBCLC or breastfeeding counselor trainer.

Abbreviations: BF, breastfeeding; IBCLC, International Board Certified Lactation Consultant.

### 3.5 Knowledge and Skills in Association With Attitudes and Experience of the Need for Additional Training on the Subject

Knowledge and skills, and attitudes revealed a high correlation (*r* = 0.523, *p* < 0.001). The respondents with better knowledge and skills had more positive attitudes toward long‐term breastfeeding. In addition, knowledge and skills, and experience of the need for additional training were statistically associated (OR = 1.95, CI 1.14−3.34, *p* = 0.015) as the respondents with better knowledge and skills were more likely to experience the need for additional training. No statistically significant association was found between attitudes and experience of the need for additional training (OR = 1.11, CI 0.61−1.96, *p* = 0.753).

## 4 Discussion

This study explored public health nurses’ competence in long‐term breastfeeding and their experience of the need for additional training on the subject in the context of Finnish maternity and child health clinics. As the previous research on the subject is lacking in Finland and limited globally, the results of this study are discussed in the wider context of high‐income countries. The results follow previous international findings in that the health professionals’ attitudes toward breastfeeding beyond 12 months grew more negative as the child gets older (Cockerham‐Colas, Geer, and Benker 2012; Rempel & McCleary 2012; Colaceci et al. 2020; Goldbort, Hitt, and Zhuang 2023). In our study, the acceptability of breastfeeding decreased concurrently with an increase in embarrassment experienced by the nurses when seeing a mother breastfeed an older child. In addition, the public health nurses’ intention to encourage the mother to stop breastfeeding increased. However, the baseline attitudes of public health nurses were predominantly positive toward breastfeeding beyond 12 months and their knowledge and skills were mainly at a good level. These results differ somewhat from previous results (Cockerham‐Colas, Geer, and Benker 2012; Baranowska et al. 2019; Zhuang et al. 2020), which have emphasized more negativity in attitudes and an insufficient level of knowledge. A culture promoting breastfeeding in the Nordic countries can be seen as an influencing factor in this context. Because of this, the results cannot be directly generalized to other countries.

Although the respondents had mainly a good level of knowledge and skills, there was a visible lack of knowledge related to night‐time breastfeeding, the prevalence of long‐term breastfeeding, and the economic aspects of breastfeeding beyond 12 months. Most of the respondents (58.1%) felt that mothers should stop night‐time breastfeeding when the child reaches the age of one. Previous studies have shown that health professionals may advise against night‐time breastfeeding (Dowling and Brown 2013) and put pressure on mothers to stop breastfeeding at night against their will (Blixt, Johansson, and Hildingsson 2019). Mothers feel that health professionals have a negative attitude toward night‐time breastfeeding (Ojantausta and Kaunonen 2022) and find that health professionals are providing misinformation about the effects of night‐time breastfeeding on oral health (Blixt, Johansson, and Hildingsson 2019). From a dental health perspective, when guiding continued breastfeeding beyond the age of one, it is important to consider limiting night‐time feedings to 1−2 times and ensuring good oral healthcare for the child (Hakulinen, Otronen, and Kuronen 2017), as recommended by the Finnish Institute for Health and Welfare. In addition to providing evidence‐based information, public health nurses should respect the wishes and decisions of families.

Respondents lacked information about the statistics of breastfeeding, as only 16% of the respondents knew that 58% of 12‐month‐old children are still breastfed in Finland. The incorrect response options in the questionnaire were 25% and 38% so this result suggests that public health nurses believe that 1‐year‐old children are breastfed less than they actually are. According to mothers' experiences, public health nurses may assume that breastfeeding ends when the child reaches the age of one, and breastfeeding is no longer discussed in healthcare settings after this point (Dowling and Brown 2013; Ojantausta and Kaunonen 2022). The lack of knowledge regarding the prevalence of breastfeeding a 1‐year‐old could also be explained by the possibility that mothers may conceal breastfeeding beyond 1 year because they fear the reaction of health professionals (Dowling and Pontin 2017; Ojantausta and Kaunonen 2022).

Further, the economical aspect of breastfeeding is quite abstract and far removed from the practicality of breastfeeding guidance and may therefore pose a challenge to health professionals’ understanding of the topic. Less than half (43.7%) of the respondents felt that breastfeeding beyond 12 months was economically beneficial to the family. However, optimal breastfeeding provides economic benefits to both families and society in general (Walters, Phan, and Mathisen 2019). Long‐term breastfeeding is economical for families due to nutritional value aspects, as breastmilk can meet a third of a child's daily nutritional needs for children aged over 1 year (Lackey et al. 2021). In the family's daily life, the benefits of long‐term breastfeeding are evident in a lower incidence of childhood infections (Victora et al. 2016), leading to economic benefits through reduced healthcare costs and fewer absences from work for the parents. Based on mothers' experiences, along with the emotional, nutritional, and health benefits of breastfeeding, the economical aspect of breastfeeding emerges as one of the significant reasons to continue breastfeeding beyond 12 months (Keim, Tchaconas, and Adesman 2017).

In our study, public health nurses’ better competence was associated with their younger age, parenthood, an additional degree in midwifery, and breastfeeding specialist certification. Respondents’ younger age was associated with both better knowledge and skills and more positive attitudes toward long‐term breastfeeding. The finding that older public health nurses are more likely to have an insufficient level of knowledge and skills, and less positive attitudes differs from previous studies, where age had no such significant effect (Baranowska et al. 2019; Zhuang, Hitt, and Goldbort 2020). However, mothers’ experiences support this age‐related finding, as in a previous study, the mothers felt that older public health nurses had a more negative attitude toward long‐term breastfeeding compared to their younger colleagues (Ojantausta and Kaunonen 2022).

Breastfeeding guidance competence is influenced by health professionals’ personal experiences and perspectives on the subject, and professionals with breastfeeding experience of their own may be stronger advocates for breastfeeding (Feldman‐Winter, Schanler, and O'Connor 2008). In our study, having a child of one's own was associated with better knowledge and skill levels. Previous studies have found an association between health professionals’ own breastfeeding experience and better knowledge level (Baranowska et al. 2019; Bowdler, Nielsen, and Moroney 2022), which is in line with our results, as 99.1% of the nurses who were parents had breastfed their child. No association was found between parenthood and attitudes in this study, which differs from previous findings (Cockerham‐Colas, Geer, and Benker 2012; Baranowska et al. 2019). In the Finnish context, this can be explained by the general breastfeeding‐friendly atmosphere in society, which also lays the foundation for the breastfeeding‐promoting approach used in maternity and child health clinics.

The respondents’ additional professional education increased their competence. Public health nurses with breastfeeding specialist certification had better knowledge and skills, and an additional degree in midwifery was related to more positive attitudes. Previous studies have also found an association between the midwife profession and more positive attitudes (Baranowska et al. 2019), as well as a higher likelihood of advising mothers to continue breastfeeding for 2 years and beyond (Shaw and Devgan 2018). These results may be linked to respondents’ education and its broader content regarding breastfeeding competence, taking into account their dual degrees as midwives‐public health nurses, as well as the diverse encounters related to breastfeeding with families across various healthcare settings. In addition, the expertise of midwives is more strongly focused on supporting families with babies compared to public health nurses, whose client groups are more varied, from school healthcare to occupational healthcare.

Based on previous research, Finnish health professionals identify a lack of knowledge and skills and negative attitudes as the major barriers to breastfeeding support in healthcare facilities (Laanterä, Pölkki, and Pietilä 2011). To provide an adequate level of support and guidance for families, health professionals need to be offered training and education (Gavine, MacGillivray, and Renfrew 2016). In our study, 72.2% of the respondents reported a need for additional training related to breastfeeding beyond 12 months. Two groups of respondents were more likely to experience the need for additional training: younger public health nurses and public health nurses with breastfeeding specialist certification. In addition, experiencing the need for additional training on the subject was, somewhat contradictorily, statistically associated with better knowledge and skills. According to our findings, a good knowledge level is required to notice a need for education, which suggests that it may not be easy to perceive a lack in one's competence. Health professionals’ breastfeeding competence goes beyond concrete counseling skills: it also requires recognizing the limits of one's knowledge and skills (USBC 2010).

Despite Finland's breastfeeding‐friendly stance, our findings had many similarities with earlier studies, which were mostly conducted in the United States and the United Kingdom. This supports the examination of long‐term breastfeeding as a broader phenomenon across high‐income countries. Although cultural attitudes toward breastfeeding an infant may vary between countries, long‐term breastfeeding is a less understood phenomenon across high‐income countries, which may reflect in the competence of health professionals.

### 4.1 Limitations

In this study, data were collected using an electronic survey questionnaire. In a self‐selected sample, the topic of the study may influence who chooses to participate and as a result, those who have a personal interest in the topic may have responded. As long‐term breastfeeding is a subject that evokes emotions and opinions, a self‐selected sample may introduce bias into the results.

Since the study was conducted in a breastfeeding‐friendly Nordic country, the results cannot be directly generalized to other countries. This can be seen in the results of this study, as the public health nurses had better knowledge and skills as well as more positive attitudes toward long‐term breastfeeding compared to previous studies on the subject.

## 5 Conclusions

This study and the evidence of previous research clearly suggest that there is a connection between health professionals’ breastfeeding‐related knowledge level and attitudes (Vandewark 2014; Baranowska et al. 2019). This link between better knowledge and more positive attitudes highlights the importance of breastfeeding education. As the personal experiences, attitudes and beliefs of health professionals may influence the breastfeeding guidance they provide to families and its evidence‐based nature (Wright and Hurst 2018), it is important that professionals understand the impact of their personal values and perceptions on the breastfeeding support they give to families. More research on the topic is needed in the Finnish context to gain a better understanding of the role of long‐term breastfeeding in Finnish healthcare and society.

### 5.1 Relevance to Clinical Practice

The results of this study indicate that long‐term breastfeeding related education and training is essential and necessary. The training should be provided regularly throughout the nurses’ working years, as older public health nurses were found to have poorer knowledge and skills level, and more negative attitudes compared to those at the beginning of their careers. The majority of public health nurses expressed a need for further training on the subject, which emphasizes the importance of education even more.

## Author Contributions

Study design: O.O., N.P., M.K., R.I. Data collection: O.O., N.P. Data analysis/interpretation: O.O., N.P., H.H., R.I. Drafting of the manuscript: O.O., N.P., H.H. Critical revision: O.O., N.P., M.K., H.H., R.I. Final approval: O.O., N.P., M.K., H.H., R.I.

## Conflicts of Interest

The authors declare no conflicts of interest.

## Supporting information



Supporting Information

## Data Availability

Research data are not shared.
