# Supplementary material for: Public Health Nurses’ Competence Related to Long‐Term Breastfeeding in the Context of Maternity and Child Health Clinics
Source: Public Health Nurs. 2024 Oct 16;42(1):221–32. doi: 10.1111/phn.13457 (PMC11700924; doi:10.1111/phn.13457)
Supplement: Supplementary file 1 — Supporting Information [file PHN-42-221-s001.docx]

**SUPPLEMENTARY MATERIAL**

Distribution of the outcome variables


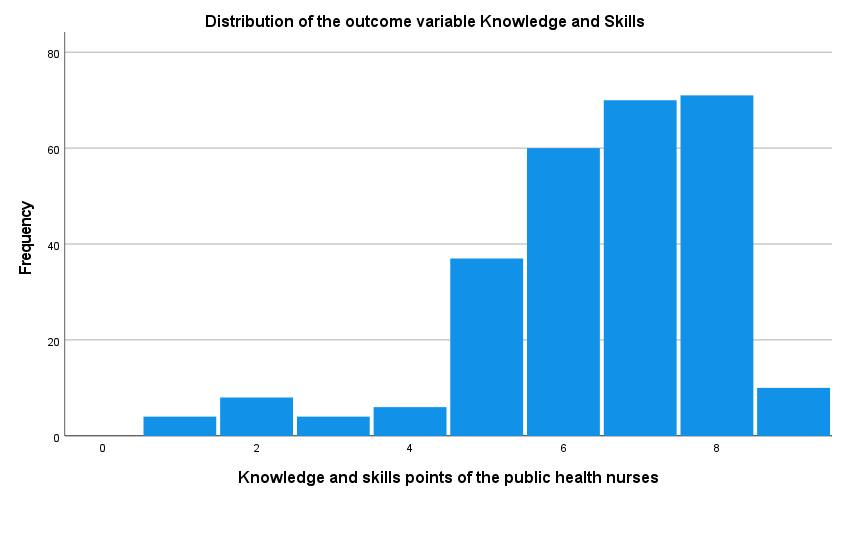


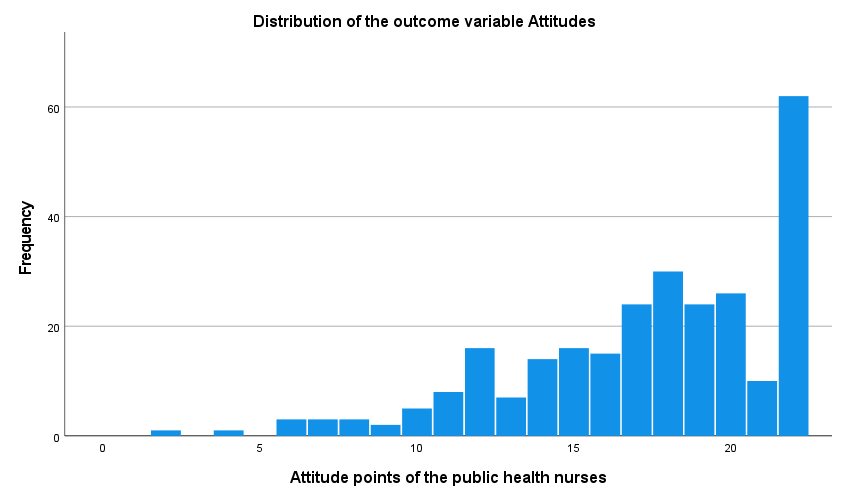


**SUPPLEMENTARY MATERIAL**

Structure of the LBCS

| **Original item** | **English translation** | **Response options** | **Origin of the item** |
| --- | --- | --- | --- |
| Suomessa vuoden ikää lähestyvistä lapsista imetettyjä on ___. | In Finland, there are ___ children approaching the age of one who are breastfed. | 25% / 38% / **58%** | Cockerham-Colas et al., 2012; Ikonen et al., 2020 |
| Äidin tulisi lopettaa vanhemman lapsen imetys raskaaksi tultuaan. | The mother should stop breastfeeding the older child after becoming pregnant. | True / **False** / Don’t know | Cockerham-Colas et al., 2012 |
| Äidin tulisi lopettaa yöaikainen imetys lapsen täytettyä vuoden. | The mother should stop nighttime breastfeeding when the child reaches one year of age. | True / **False** / Don’t know | Dowling & Brown, 2013; Blixt et al., 2019; Ojantausta & Kaunonen, 2022 |
| Suomessa suositellaan imetyksen lopettamista, kun lapsi täyttää vuoden. | In Finland, it is recommended to stop breastfeeding when the child reaches one year of age. | True / **False** / Don’t know | Cockerham-Colas et al., 2012; Finnish Institute for Health and Welfare, 2019 |
| Yli 1-vuotiaan imetys on perheelle taloudellisesti hyödyllistä. | Breastfeeding beyond 12 months is economically beneficial for the family. | **True** / False / Don’t know | Zhuang et al., 2020; Ojantausta & Kaunonen, 2022 |
| Rintamaito on hyvää ravintoa yli 1-vuotiaalle lapselle. | Breast milk is good nutrition for a child over 1 year old. | **True** / False / Don’t know | Dewey, 2001; Zhuang et al., 2020 |
| Yli vuoden imettäneillä naisilla ennen vaihdevuosia ilmenevän rintasyövän esiintyvyys on ___. | Women who have breastfed for more than a year have a___ risk of premenopausal breast cancer. | Higher / **Lower** / Don’t know | Cockerham-Colas et al., 2012 |
| Yli vuoden imetetyillä lapsilla lapsuus- ja aikuisiän lihavuuden riski on ___. | Children breastfed for more than a year have a ___ risk of childhood and adult obesity. | Higher / **Lower** / Don’t know | Victora et al., 2016, Baranowska et al., 2019, Zhuang et al., 2020, Ojantausta & Kaunonen, 2021 |
| Yli 1-vuotiaan lapsen imetyksen vaikutus vanhemman ja lapsen väliseen kiintymyssuhteeseen on ___. | The effect of breastfeeding beyond 12 months on the attachment relationship between parent and child is ___. | **Positive** / Negative / Neutral | Baranowska et al., 2019; Zhuang et al., 2020; Ojantausta & Kaunonen, 2021 |
| Mielestäni yli 1-vuotiaan lapsen imettäminen on luonnollista. | I think that breastfeeding a child beyond 1 year of age is normal. | 5-point Likert scale* | Cockerham-Colas et al., 2012 |
| Mielestäni on hyväksyttävää, että äiti imettää 1-vuotiasta lasta. | I think that it is acceptable for 1-year-old children to be breastfed by their mothers. | 5-point Likert scale* | Cockerham-Colas et al., 2012 |
| Mielestäni on hyväksyttävää, että äiti imettää 2-vuotiasta lasta. | I think that it is acceptable for 2-year-old children to be breastfed by their mothers. | 5-point Likert scale* | Cockerham-Colas et al., 2012 |
| Mielestäni on hyväksyttävää, että äiti imettää 3-vuotiasta tai vanhempaa lasta. | I think that it is acceptable for 3-year-old or older children to be breastfed by their mothers. | 5-point Likert scale* | Cockerham-Colas et al., 2012 |
| Olisin kiusaantunut, jos äiti imettäisi läsnäollessani 1-vuotiasta lasta. | I would be embarrassed if a mother breastfed her 1-year-old child in front of me. | 5-point Likert scale* | Cockerham-Colas et al., 2012 |
| Olisin kiusaantunut, jos äiti imettäisi läsnäollessani 2-vuotiasta lasta. | I would be embarrassed if a mother breastfed her 2-year-old child in front of me. | 5-point Likert scale* | Cockerham-Colas et al., 2012 |
| Olisin kiusaantunut, jos äiti imettäisi läsnäollessani 3-vuotiasta tai vanhempaa lasta. | I would be embarrassed if a mother breastfed her child of 3 years or older in front of me. | 5-point Likert scale* | Cockerham-Colas et al., 2012 |
| Kehottaisin 1-vuotiasta lasta imettävää äitiä lopettamaan imetyksen. | I would encourage a woman breastfeeding her 1-year-old child to wean. | 5-point Likert scale* | Cockerham-Colas et al., 2012 |
| Kehottaisin 2-vuotiasta lasta imettävää äitiä lopettamaan imetyksen. | I would encourage a woman breastfeeding her 2-year-old child to wean. | 5-point Likert scale* | Cockerham-Colas et al., 2012 |
| Kehottaisin 3-vuotiasta lasta imettävää äitiä lopettamaan imetyksen. | I would encourage a woman breastfeeding her 3-year-old or older child to wean. | 5-point Likert scale* | Cockerham-Colas et al., 2012 |
| 1-vuotiaan lapsen imetyksellä on fyysisiä terveyshyötyjä lapselle. | In general, I think breastfeeding a 1-year-old child would benefit a child’s physical health. | 5-point Likert scale* | Cockerham-Colas et al., 2012 |
| 2-vuotiaan lapsen imetyksellä on fyysisiä terveyshyötyjä lapselle. | In general, I think breastfeeding a 2-year-old child would benefit a child’s physical health. | 5-point Likert scale* | Cockerham-Colas et al., 2012 |
| 3-vuotiaan tai vanhemman lapsen imetyksellä on fyysisiä terveyshyötyjä lapselle. | In general, I think breastfeeding a child of 3 years or older would benefit a child’s physical health | 5-point Likert scale* | Cockerham-Colas et al., 2012 |
| 1-vuotiaan lapsen imettäminen voi aiheuttaa lapselle psyykkistä haittaa. | In general, I think breastfeeding a 1-year-old child could cause psychological harm to the child. | 5-point Likert scale* | Cockerham-Colas et al., 2012 |
| 2-vuotiaan lapsen imettäminen voi aiheuttaa lapselle psyykkistä haittaa. | In general, I think breastfeeding a 2-year-old child could cause psychological harm to the child. | 5-point Likert scale* | Cockerham-Colas et al., 2012 |
| 3-vuotiaan tai vanhemman lapsen imettäminen voi aiheuttaa lapselle psyykkistä haittaa. | In general, I think breastfeeding a child of 3 years or older could cause psychological harm to the child. | 5-point Likert scale* | Cockerham-Colas et al., 2012 |
| 1-vuotiaan lapsen imetyksellä on fyysisiä terveyshyötyjä äidille. | In general, I think breastfeeding a 1-year-old child would benefit a mother’s physical health. | 5-point Likert scale* | Cockerham-Colas et al., 2012 |
| 2-vuotiaan lapsen imetyksellä on fyysisiä terveyshyötyjä äidille. | In general, I think breastfeeding a 2-year-old child would benefit a mother’s physical health. | 5-point Likert scale* | Cockerham-Colas et al., 2012 |
| 3-vuotiaan tai vanhemman lapsen imetyksellä on fyysisiä terveyshyötyjä äidille. | In general, I think breastfeeding a child of 3 years or older would benefit a mother’s physical health. | 5-point Likert scale* | Cockerham-Colas et al., 2012 |
| 1-vuotiaan lapsen imettäminen voi aiheuttaa äidille psyykkistä haittaa. | In general, I think breastfeeding a 1-year-old child could cause psychological harm to the mother. | 5-point Likert scale* | Cockerham-Colas et al., 2012 |
| 2-vuotiaan lapsen imettäminen voi aiheuttaa äidille psyykkistä haittaa. | In general, I think breastfeeding a 2-year-old child could cause psychological harm to the mother. | 5-point Likert scale* | Cockerham-Colas et al., 2012 |
| 3-vuotiaan tai vanhemman lapsen imettäminen voi aiheuttaa äidille psyykkistä haittaa. | In general, I think breastfeeding a child of 3 years or older could cause psychological harm to the mother. | 5-point Likert scale* | Cockerham-Colas et al., 2012 |

* Completely agree, Somewhat agree, Neither agree nor disagree, Somewhat disagree, Completely disagree
